# Trilaminar DuraGen-Anchored Closure for Dural Repair in Posterior Fossa Surgery: Technical Note and Case Series

**DOI:** 10.1227/neuprac.0000000000000204

**Published:** 2026-02-02

**Authors:** Young Ju Kim, Masashi Higashino, Shunsuke Yamanishi, Noriaki Ashida, Kohkichi Hosoda, Hiroaki Nagashima, Kazuhiro Tanaka, Takashi Sasayama, Masamitsu Nishihara

**Affiliations:** 1 Department of Neurosurgery, Nishikobe Medical Center, Kobe, Hyogo, Japan;; 2 Department of Neurosurgery, Kosei Hospital, Kobe, Hyogo, Japan;; 3 Department of Neurosurgery, Myodani Hospital, Kobe, Hyogo, Japan;; 4 Department of Neurosurgery, Kobe University Graduate School of Medicine, Kobe, Hyogo, Japan

**Keywords:** Cerebrospinal fluid leak, Duraplasty, DuraGen, Posterior fossa, Technical note, Trilaminar closure

## Abstract

**BACKGROUND AND OBJECTIVES:**

Cerebrospinal fluid (CSF) leakage remains a common complication of posterior fossa surgery, often exacerbated by early postoperative mobilization. We describe a novel fibrin glue-free technique—trilaminar DuraGen-anchored closure—designed to enhance watertight dural sealing through circumferential suturing of DuraGen patches placed both subdurally and epidurally.

**METHODS:**

We retrospectively reviewed 20 patients who underwent posterior fossa surgery with this closure technique between 2020 and 2025. A subdural DuraGen patch approximately 1 cm larger than the dural defect was sutured circumferentially to the native dura. A second DuraGen layer was placed epidurally and similarly sutured. Although fibrin glue was used to seal exposed mastoid air cells, it was deliberately omitted from the duraplasty itself. CSF leak and wound-related complications were assessed clinically, although pseudomeningocele was evaluated radiologically using noncontrast computed tomography on postoperative day 1 and magnetic resonance imaging within 3 months.

**RESULTS:**

MRI follow-up was obtained in 18 of 20 patients (90%), with a mean interval of 20.6 months (median: 18.5 months; interquartile range: 16.1 months). The 2 patients without MRI underwent computed tomography and clinical follow-up, with no complications observed. No CSF leaks, pseudomeningoceles, or wound-related complications were identified. No reoperations or lumbar drainage were required. The technique was straightforward and reproducible across levels of surgical experience.

**CONCLUSION:**

The trilaminar DuraGen-anchored closure is a simple and effective fibrin glue-free method for achieving watertight dural closure in posterior fossa surgery. Its consistent performance supports further evaluation in broader clinical settings.

Dural closure in posterior fossa surgery presents a unique technical challenge because of anatomic constraints, gravitational cerebrospinal fluid (CSF) pooling, and the proximity of mastoid air cells.^1,2^ Inadequate dural sealing in this region increases the risk of CSF leak and pseudomeningocele formation, necessitating reliable closure strategies. DuraGen (Integra LifeSciences) is an absorbable dural substitute composed of purified type I bovine collagen. It provides a porous scaffold that promotes fibroblast infiltration and neodura formation, supporting effective dural repair. DuraGen is absorbable, easy to handle, and often used as an onlay graft without the need for sutures or fibrin glue. However, under high CSF pressure conditions, traditional onlay methods using DuraGen and fibrin glue may fail to provide a watertight seal.

Multiple studies have demonstrated that dural repair using suturable grafts and/or sealants provides more robust watertight closure than onlay grafting alone, particularly in posterior fossa procedures. Techniques such as fascia lata button duroplasty or collagen-based inlay/onlay combinations with suture fixation have shown improved mechanical integrity and reduced CSF leak rates.^2,3^ However, systematic reviews have also suggested that sealants alone may not significantly reduce CSF leak incidence, although they may lower surgical site infection rates.^1,2,4,5^

CSF leakage remains one of the most common complications of posterior fossa surgery, with incidence rates reported between 0.3% and 33.3%, depending on closure technique and patient factors.^2^ For instance, Inoue et al^2^ reported CSF leak rates of 5.0% using autologous fascia and 3.3% using a double-layer (inlay and onlay) DuraGen technique in microvascular decompression. In their approach, the inlay DuraGen graft was sutured to the dural edge and fixed with fibrin glue, whereas the onlay graft relied on apposition and glue without suturing. Although their results suggest noninferiority of collagen matrix closure, the lack of structural anchoring may limit effectiveness under high-pressure conditions.

To address these limitations, we present a novel suturing technique—trilaminar DuraGen-anchored closure—which enhances closure integrity in posterior fossa surgery by anchoring both subdural and epidural DuraGen patches directly to the native dura, thus obviating the need for fibrin glue during duraplasty (Figure 1).

**FIGURE 1.**
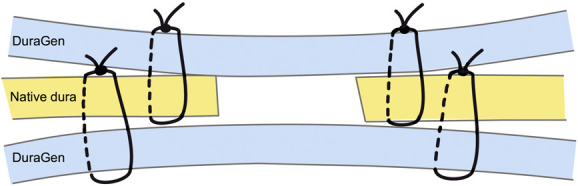
Schematic representation of the trilaminar DuraGen-anchored closure technique. Subdural and epidural DuraGen patches are sutured to the native dura.

## METHODS

### Study Design and Setting

We conducted a retrospective case series of patients who underwent posterior fossa surgery using the trilaminar DuraGen-anchored dural closure technique between January 2020 and March 2025 at a single academic institution. Eligible patients were adults aged 18 years or older who underwent either a retrosigmoid or midline suboccipital craniotomy, and in whom dural closure was performed using the described trilaminar technique. All surgeries were performed by the same senior neurosurgeon.

### Surgical Technique

**FIGURE 2.**
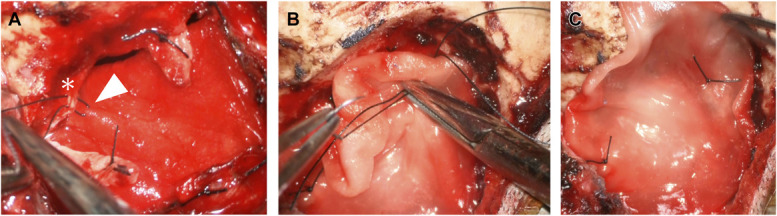
Intraoperative views. **A**, Subdural DuraGen (in-layer) placement. The inlay DuraGen is approximately 1 cm larger than the dural defect and is circumferentially tucked beneath the native dura to enhance contact and mechanical stability. The asterisk marks the native dura. Interrupted sutures are placed approximately 0.5 cm apart to secure the inlay. The white arrowhead indicates the needle passed through both the native dura and the in-layer DuraGen immediately before ligation. **B**, Epidural DuraGen (on-layer) anchoring. **C**, Final closure.

After retrosigmoid or midline suboccipital craniotomy, a skin–galea–periosteal flap is elevated, and the dura is opened cruciately. A DuraGen patch approximately 1 cm larger than the dural defect is inserted subdurally and sutured circumferentially, sutured using 4-0 Nurolon simple stitches approximately 5 mm apart (Figure 2A). A second DuraGen patch is placed epidurally and similarly sutured at 2 to 3 fixation points (Figure 2B and 2C,). No fibrin glue is used for duraplasty itself. Bone fragments are reattached with fibrin glue; mastoid air cells are sealed using autologous muscle and glue. No fibrin glue is applied to the duraplasty.

### Outcome Measures

Postoperative evaluation included both clinical and radiographic assessments. Clinically, all patients were examined for signs of CSF otorrhea and incisional CSF leakage. Radiographically, noncontrast computed tomography (CT) was performed on postoperative day 1 in all patients. MRI follow-up was scheduled within 3 months after surgery to evaluate early pseudomeningocele formation, and long-term MRI follow-up was additionally obtained when available.

All imaging studies were independently reviewed by the operating neurosurgeon and a radiologist. Primary outcomes included the presence of CSF leak or pseudomeningocele as determined by clinical signs or imaging findings. Secondary outcomes included wound-related complications such as dehiscence or infection, and the need for reoperation or lumbar drainage.

### Ethical Considerations

This study was conducted in accordance with institutional guidelines and was approved as exempt from full institutional review board approval, as all procedures represented routine clinical care without deviation from standard practice. DuraGen was appropriately used within the framework of the Japanese national health insurance system, and its cost was properly charged to the patient in compliance with institutional and national billing regulations. Written informed consent for data use and video documentation was obtained from all patients before surgery. Although the suturing configuration represents a novel adaptation, it was implemented within standard duraplasty practice and therefore qualified for exempt review.

## RESULTS

### Patient Cohort

Twenty consecutive patients (7 male and 13 female; median age 69, range 38-85 years) underwent posterior fossa surgery using the trilaminar DuraGen-anchored closure technique between 2020 and 2025. Indications included vestibular schwannoma (n = 7), hemangioblastoma (n = 3), meningioma (n = 2), malignant glioma (n = 2), and 6 other pathologies (see Table for full list).

**TABLE.** Patient Demographics and Surgical Outcomes

Patient no.	Age (y)	Sex	Diagnosis	Op approach	Postoperative CSF leak	Other complications
1	59	F	Vagal schwannoma	Retrosigmoid	None	None
2	40	F	Trigeminal neuralgia	Retrosigmoid	None	None
3	55	M	Vestibular schwannoma	Retrosigmoid	None	None
4	38	M	Vestibular schwannoma	Retrosigmoid	None	None
5	70	M	Hemangioblastoma	Retrosigmoid	None	None
6	45	F	Malignant glioma	Retrosigmoid	None	None
7	75	F	Glossopharyngeal neuralgia	Retrosigmoid	None	None
8	68	F	Vestibular schwannoma	Retrosigmoid	None	None
9	69	F	Meningioma	Retrosigmoid	None	None
10	79	F	Malignant glioma	Midline suboccipital	None	None
11	85	F	Metastasis	Retrosigmoid	None	None
12	76	F	Trigeminal schwannoma	Retrosigmoid	None	None
13	64	F	Lymphoid aggregation	Retrosigmoid	None	None
14	78	F	Vestibular schwannoma	Retrosigmoid	None	None
15	69	F	Meningioma	Retrosigmoid	None	None
16	71	M	Hemangioblastoma	Retrosigmoid	None	None
17	75	M	Vestibular schwannoma	Retrosigmoid	None	None
18	39	M	Hemangioblastoma	Midline suboccipital	None	None
19	80	F	Vestibular schwannoma	Retrosigmoid	None	None
20	61	F	Vestibular schwannoma	Midline suboccipital	None	None

Additional demographics: mean body mass index was 22.19 kg/m^2^ (median 22.26; IQR 20.56-24.19). Two patients had diabetes mellitus.

CSF, cerebrospinal fluid; F, female; Op, operative; M, male.

Postoperative CSF leak indicates clinically or radiologically confirmed CSF leakage following surgery.

Other complications refers to any additional adverse events noted during the postoperative period.

**FIGURE 3.**
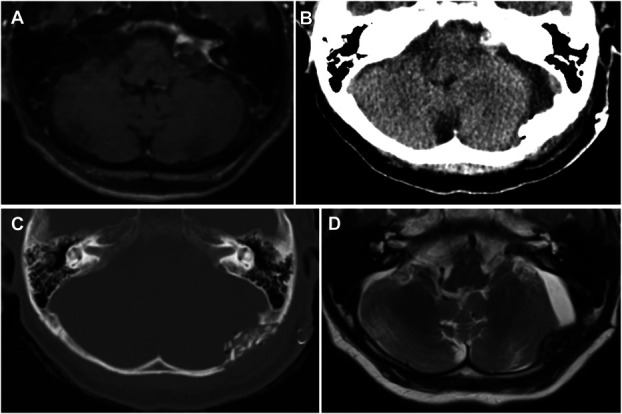
Postoperative imaging of Patient No. 19 with a left vestibular schwannoma, demonstrating no CSF leakage. **A**, Preoperative gadolinium-enhanced axial MRI showing a contrast-enhancing tumor in the left cerebellopontine angle. **B**, Noncontrast computed tomography on postoperative day 1 without evidence of CSF leakage. **C**, Bone window computed tomography on postoperative day 1 showing no abnormal findings. **D**, T2-weighted MRI on postoperative day 76 confirming absence of CSF leakage. CSF, cerebrospinal fluid.

CSF leakage was assessed clinically, by CT on postoperative day 1, and by MRI within 3 months of surgery (Figure 3). In addition, long-term MRI follow-up was obtained in 18 of 20 patients (90%), with a mean interval of 20.6 months (median: 18.5 months; IQR: 16.1 months). The 2 patients without long-term MRI underwent CT and clinical follow-up, with no evidence of CSF leakage or wound-related complications.

All patients tolerated early ambulation beginning on postoperative day 1. None of the 20 patients developed CSF leakage, pseudomeningocele, or wound-related complications. No reoperations or lumbar drainage procedures were required.

## DISCUSSION

Posterior fossa duraplasty presents unique technical challenges because of the confined surgical corridor, gravitational CSF pooling, and the anatomic proximity of mastoid air cells. Effective closure in this region requires a balance between mechanical strength and material conformability. Our trilaminar DuraGen-anchored closure technique was developed to address these issues by combining the handling advantages of a collagen-based matrix with the added security of circumferential suture fixation.

In this 20-patient case series, no postoperative CSF leakage, pseudomeningocele, or wound-related complications were observed. The closure maintained mechanical integrity sufficient for early mobilization and was reproducible among both senior and junior surgeons without significantly extending operative time. From a technical standpoint, the suturing was straightforward, making the technique suitable even in training settings.

Compared with previous approaches—such as double DuraGen technique described by Inoue et al,^2^ which involved suture fixation of the inlay graft with fibrin glue and apposition of the onlay layer without suturing—our method emphasizes fully circumferential suture-based fixation of both layers without the use of fibrin glue, which may enhance watertightness in anatomically high-risk regions such as the posterior fossa. This concept of suture-based mechanical reinforcement has also been applied in endoscopic skull base surgery, where fascia lata button grafts have been used to strengthen dural closure and prevent CSF leakage.^3^ Although Inoue et al's^2^ approach prioritized simplicity through limited suture use and glue-assisted fixation, our trilaminar DuraGen-anchored technique offers a more structurally reinforced closure by securing both subdural and epidural layers directly to the native dura with sutures alone. This structural anchoring may provide superior watertightness, particularly in posterior fossa surgeries, where gravitational CSF pooling and mastoid air cell exposure poses additional risk factors for leakage.

DuraGen is absorbable and readily available in Japan. It is favored for its pliability and ease of handling in confined surgical spaces such as the posterior fossa. By contrast, suturable synthetic grafts such as expanded polytetrafluoroethylene, which are occasionally used in neurosurgical duraplasty, provide greater tensile strength. However, their rigidity can reduce conformability and make handling more difficult in curved anatomic regions. Our technique uniquely applies suture-based fixation to a pliable collagen matrix, aiming to combine the benefits of ease of handling and structural security, which are often difficult to achieve simultaneously with a single material.

Furthermore, the exclusive use of sutures eliminates the need for fibrin glue in duraplasty, potentially enhancing both safety and cost-effectiveness. Although fibrin glue was used for bone flap fixation and mastoid sealing, its omission from the dural repair itself may modestly reduce overall material costs.

To date, this technique has been performed exclusively at Nishikobe Medical Center, and its consistent reproducibility within our institution supports its feasibility and reliability in a real-world neurosurgical setting.

Historical CSF leak rates at our institution were unavailable because of the variability of dural closure techniques used in the past. Therefore, this study was designed as a technical feasibility report rather than a comparative outcome analysis.

### Limitations

This study is subject to several limitations, including its retrospective nature, small sample size, and single-center setting. The absence of a control group limits the ability to make direct comparisons with other duraplasty techniques, and objective measures of watertightness—such as CSF pressure monitoring or standardized imaging—were not used. Although clinical and CT-based evaluations were performed in all cases, follow-up MRI was not obtained in 2 of 20 patients. This may limit the uniformity of radiographic assessment, particularly for detecting asymptomatic pseudomeningocele.

In addition, all cases were performed by a single surgical team, which may introduce institutional bias. As this work is presented as a technical note, its primary objective is to introduce and evaluate the feasibility and safety of a novel dural closure method. Future prospective, multicenter studies with larger cohorts are warranted to validate its broader applicability and long-term effectiveness.

## CONCLUSION

The trilaminar DuraGen-anchored closure technique is a novel, practical, and effective method for achieving watertight dural closure in posterior fossa surgery. It offers a fibrin glue-free alternative for dural closure, although fibrin glue was used selectively for mastoid air cell sealing. No postoperative complications were observed, and this technique may be broadly applicable to posterior fossa procedures requiring reliable and reproducible dural reconstruction.
